# Influence of *B. subtilis* 3NA mutations in *spo0A* and *abrB* on surfactin production in *B. subtilis* 168

**DOI:** 10.1186/s12934-021-01679-z

**Published:** 2021-09-26

**Authors:** Peter Klausmann, Lars Lilge, Moritz Aschern, Katja Hennemann, Marius Henkel, Rudolf Hausmann, Kambiz Morabbi Heravi

**Affiliations:** Institute of Food Science and Biotechnology, Department of Bioprocess Engineering (150K), University of Hohenheim, Fruwirthstraße 12, 70599 Stuttgart, Germany

**Keywords:** *Bacillus subtilis*, High cell density, Surfactin, Lipopeptide, AbrB, Spo0A, Strain engineering

## Abstract

**Background:**

*Bacillus subtilis* is a well-established host for a variety of bioproduction processes, with much interest focused on the production of biosurfactants such as the cyclic lipopeptide surfactin. Surfactin production is tightly intertwined with quorum sensing and regulatory cell differentiation processes. As previous studies have shown, a non-sporulating *B. subtilis* strain 3NA encoding a functional *sfp* locus but mutations in the *spo0A* and *abrB* loci, called JABs32, exhibits noticeably increased surfactin production capabilities. In this work, the impacts of introducing JABs32 mutations in the genes *spo0A*, *abrB* and *abh* from 3NA into strain KM1016, a surfactin-forming derivative of *B. subtilis* 168, was investigated. This study aims to show these mutations are responsible for the surfactin producing performance of strain JABs32 in fed-batch bioreactor cultivations.

**Results:**

Single and double mutant strains of *B. subtilis* KM1016 were constructed encoding gene deletions of *spo0A*, *abrB* and homologous *abh*. Furthermore, an elongated *abrB* version, called abrB*, as described for JABs32 was integrated. Single and combinatory mutant strains were analysed in respect of growth behaviour, native P_*srfA*_ promoter expression and surfactin production. Deletion of *spo0A* led to increased growth rates with lowered surfactin titers, while deletion or elongation of *abrB* resulted in lowered growth rates and high surfactin yields, compared to KM1016. The double mutant strains *B. subtilis* KM1036 and KM1020 encoding Δspo0A abrB* and Δ*spo0A* Δ*abrB* were compared to reference strain JABs32, with KM1036 exhibiting similar production parameters and impeded cell growth and surfactin production for KM1020. Bioreactor fed-batch cultivations comparing a Δspo0A abrB* mutant of KM1016, KM681, with JABs32 showed a decrease of 32% in surfactin concentration.

**Conclusions:**

The genetic differences of *B. subtilis* KM1016 and JABs32 give rise to new and improved fermentation methods through high cell density processes. Deletion of the *spo0A* locus was shown to be the reason for higher biomass concentrations. Only in combination with an elongation of *abrB* was this strain able to reach high surfactin titers of 18.27 g L^−1^ in fed-batch cultivations. This work shows, that a *B. subtilis* strain can be turned into a high cell density surfactin production strain by introduction of two mutations.

## Background

*Bacillus subtilis* is a commonly used bacterial system for the formation of industrially relevant products. Based on their capacity to serve as super-secreting cell factories [34], production of notable amounts of valuable enzymes such as proteases and lipases is feasible [17, 25]. Furthermore, *B. subtilis* is capable of forming bioactive metabolites (e.g. surfactin and fengycin), which exhibit promising properties with broad applications [14].

Although *B. subtilis* reveals several excellent capabilities for bioproduct formation, further improvements in respect of molecular strain engineering and bioprocess engineering have been achieved [6, 11]. One important aspect is based on the cell differentiation during fed-batch processes. Due to different regulatory mechanisms involved in *B. subtilis*, varying differentiations could be initiated simultaneously such as competence development and sporulation [29]. To increase cell biomass yields, Wenzel et al. [36] used the nonsporulating *B. subtilis* 3NA strain [18] which enables high cell density fermentation processes. Corresponding fed-batch fermentations were introduced to produce eGFP as an exemplary protein of interest. Genetic characterisation of *B. subtilis* 3NA strain revealed that it was a hybrid strain composed of features from *B. subtilis* 168 and W23 with several noticeable gene modifications being identified [27]. Specifically, a nonsense mutation in the *spo0A* gene was identified, which prevents the expression of a functional version of this master regulator for sporulation initiation. Additionally, a mutation of the stop codon in *abrB* gene was evident, which results in an eleven amino acids comprising C-terminal elongation of the AbrB regulator [27].

Both regulators, Spo0A and AbrB, are important switch points for cell differentiation and cell adaptation in *B. subtilis*. Spo0A plays a crucial role for the initiation of sporulation [26]. The regulator activity is controlled by a phosphorelay mechanism that activates Spo0A by phosphorylation [3]. In the active state, Spo0A-P modulates the expression of more than 120 genes [20]. A deletion or inactivation of *spo0A* gene inhibits the sporulation process resulting in non-sporulating *B. subtilis* strains [35, 36]. Moreover, due to an antagonistic effect of Spo0A on Rok repressor in respect of *comK* gene expression [19], *spo0A* deficient *B. subtilis* strains exhibit a drastically reduced competence development which is partially reversed in combination with an inactive *abrB* version [1]. In this context, Reuß et al. [27] reported about transformation frequencies for 3NA strain comparable to well-established *B. subtilis* 168.

Another target gene of Spo0A is *abrB* that is negatively affected in the gene expression when a functional Spo0A version is present [4, 24]. As a global transcriptional regulator, AbrB affects target genes in their expression which are involved in the transition from exponential to stationary growth phase. Altogether, at least 190 genes are targeted by AbrB [4]. Beyond that, the regulatory network seems to be more extensive due to an interactive role of AbrB and its homologous Abh as homomers and heteromers, respectively, with varying affinities [4]. One target is the *srfA* operon encoding for surfactin-forming non-ribosomal peptide synthetase (NRPS). Results from [4] demonstrated a stronger derepression of *srfA* operon in an *abh* deletion mutant, although only a weak Abh binding was detectable in an *abrB* deletion and AbrB binding was retained in *abh* deletion background.

In this study, surfactin-forming *B. subtilis* strain KM1016, an *sfp*^+^ derivative of *B. subtilis* 168, was used to verify the impacts of regulators Spo0A, AbrB and Abh on the surfactin production. Therefore, both *srfA* operon expression and surfactin formation were analysed in combinatory mutant strains encoding gene deletions in *spo0A*, *abrB* and *abh* as well as an *abrB* elongation as described for 3NA strain.

## Results

### Comparison of surfactin production in *B. subtilis* wild-type strains

**Fig. 1:**
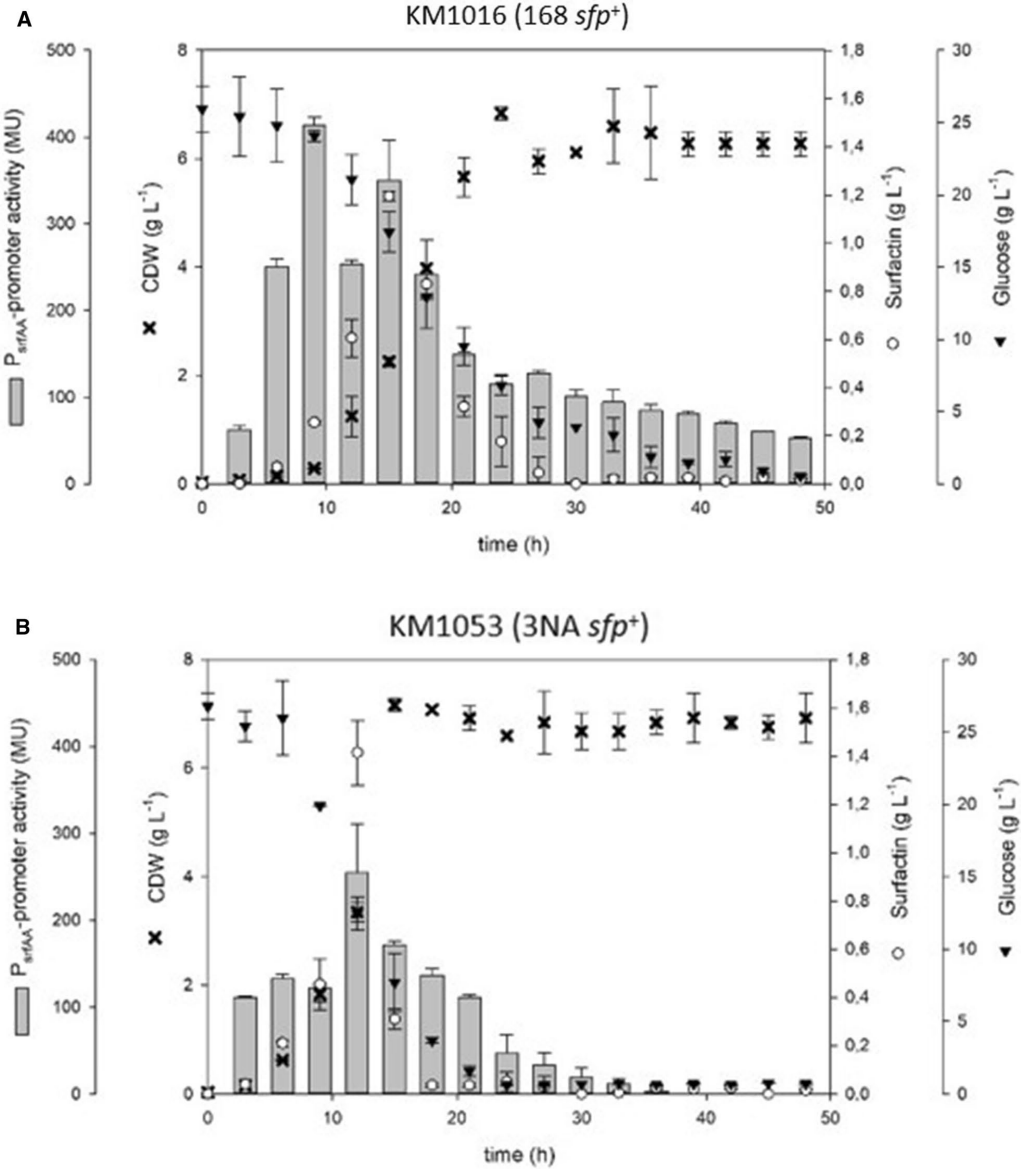
Time course of shake flask cultures of the *B. subtilis* reference strains KM1016 (168 *sfp*^+^) (**A**) and KM1053 (3NA *sfp*^+^) (**B**) displaying biomass (black crosses), surfactin (white circles) and glucose (black inverted triangles) concentrations in [g L^−1^] as well as P_*srfA*_ promoter activity (grey bars) in MU over time

Figure 1 shows shake flask cultivations of KM1053 (3NA *sfp*^+^) and KM1016 (168 *sfp*^+^). Comparison of these strains shows a significant difference in growth rates, with KM1053 at 0.28 h^−1^ and KM1016 at 0.17 h^−1^. Furthermore, the KM1053 produced about 25% more surfactin than KM1016 during the cultivation process, although a significantly lower P_*srfA*_ promoter activity (approx. 250 MU) was detectable for KM1053 compared to KM1016 (approx. 425 MU). When the maximum surfactin concentrations of 1.5 g L^−1^ and 1.2 g L^−1^, respectively, were reached after a cultivation time between 12 to 15 h, a decline of the surfactin concentration was measured for both strains. Accordingly, no surfactin could be detected after 18 h for KM1053 and after 27 h for KM1016. In this context, similar but time-delayed expression patterns were measured for the P_*srfA*_ promoter activity.

### Impact of Spo0A on surfactin production

**Fig. 2:**
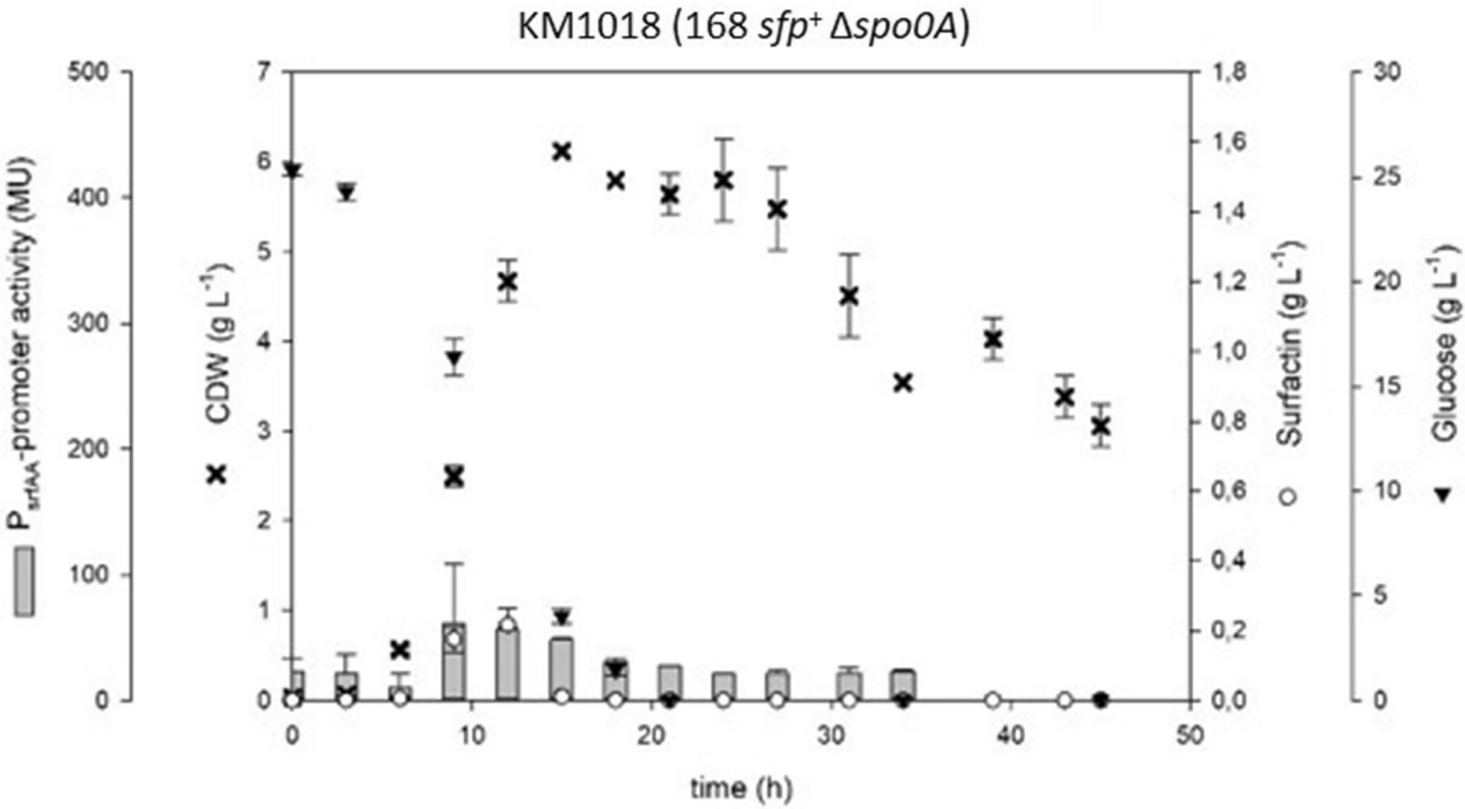
Time course of shake flask cultures of the *B. subtilis* KM1018 (168 *sfp*^+^ Δ*spo0A*) cultivation displaying biomass (black crosses), surfactin (white circles) and glucose (black inverted triangles) concentrations in [g L^−1^] as well as P_*srfA*_ promoter activity (grey bars) in MU over time

A relevant difference between *B. subtilis* derivatives of 168 and 3NA is the presence of a nonsense mutation in *spo0A* gene in 3NA [27]. To verify the influence of Spo0A on surfactin production, a KM1016 strain encoding Δ*spo0A* deletion, called KM1018 (168 *sfp*^+^ Δ*spo0A*), was constructed. Figure 2 shows growth behavior and growth rates comparable to KM1053 (3NA *sfp*^+^) but significant reductions in P_*srfA*_ promoter activity (70 MU) as well as in surfactin production capabilities, with a maximum of 0.2 g L^−1^ and rapidly decreasing concentrations after 12 h. Table 1 shows important parameters of this strain in comparison to other strains of this study.

### Impact of AbrB and its elongation on surfactin production

**Fig. 3:**
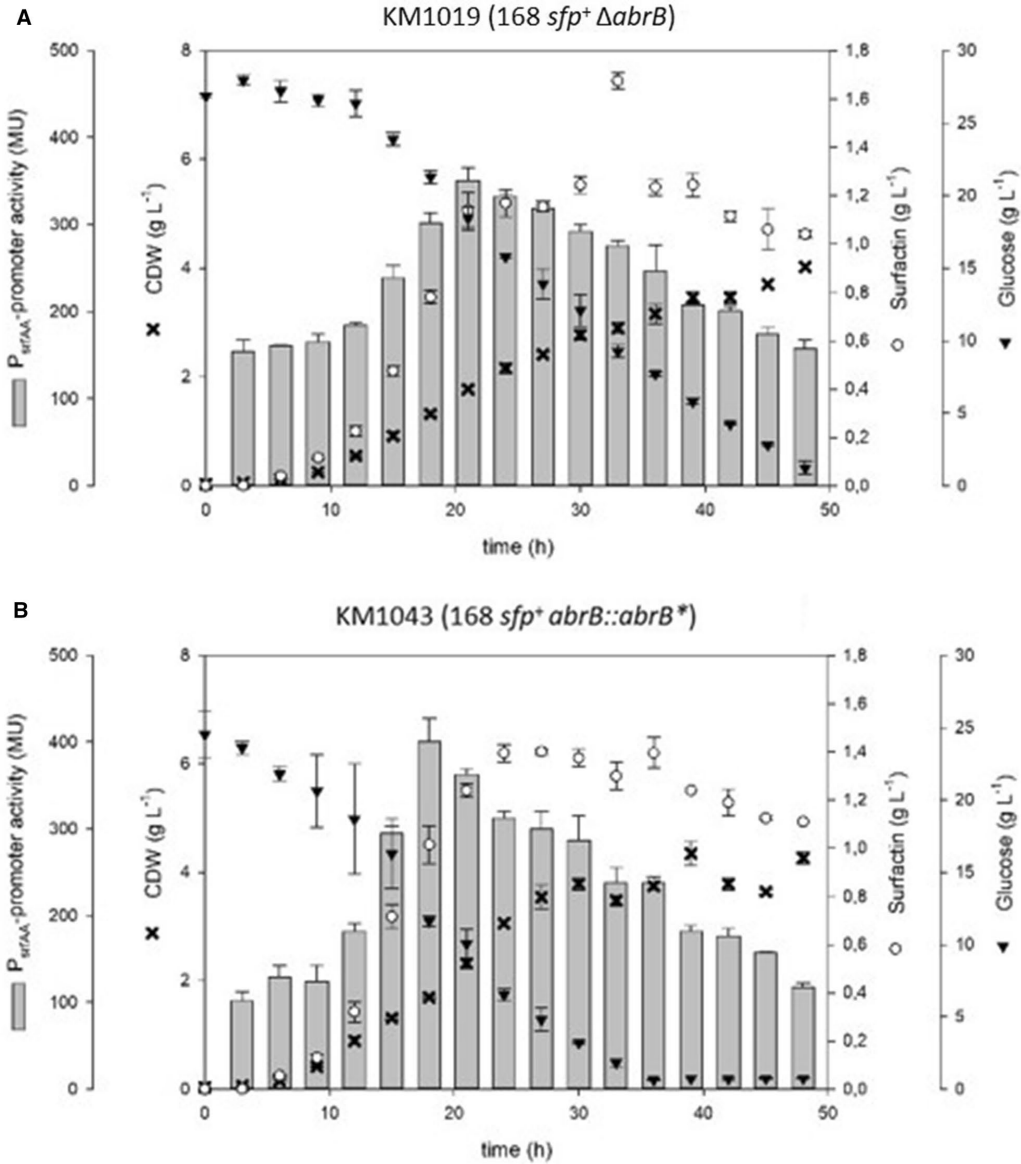
Time course of shake flask cultures of *B. subtilis* KM1019 (168 *sfp*^+^ Δ*abrB*) (**A**) and KM1043 (168 *sfp*^+^ *abrB*::abrB*) (**B**) displaying biomass (black crosses), surfactin (white circles) and glucose (black inverted triangles) concentrations in [g L^−1^] as well as P_*srfA*_ promoter activity (grey bars) in MU over time

Another significant variation between *B. subtilis* strain 168 and 3NA is the inclusion of an elongation region (33 bp) associated with the 3NA *abrB* locus, designated abrB* [27]. Effects on 168 derivative strain KM1016 were analysed by *abrB* deletion (KM1019; 168 *sfp*^+^ Δ*abrB*) (Fig. 3A) and *abrB* elongation (KM1043; 168 *sfp*^+^ *abrB*::abrB*) (Fig. 3B) as described for 3NA strain. Both strains exhibited low growth rates of 0.08 h^−1^ for KM1019 and KM1043. With lower maximum CDWs compared to the reference strain KM1016, these strains still matched its surfactin producing capabilities. Promoter activity also exhibited comparative maximum values to KM1016 as shown in Table 1.

### Influence of AbrB homolog, Abh, on surfactin production

**Fig. 4:**
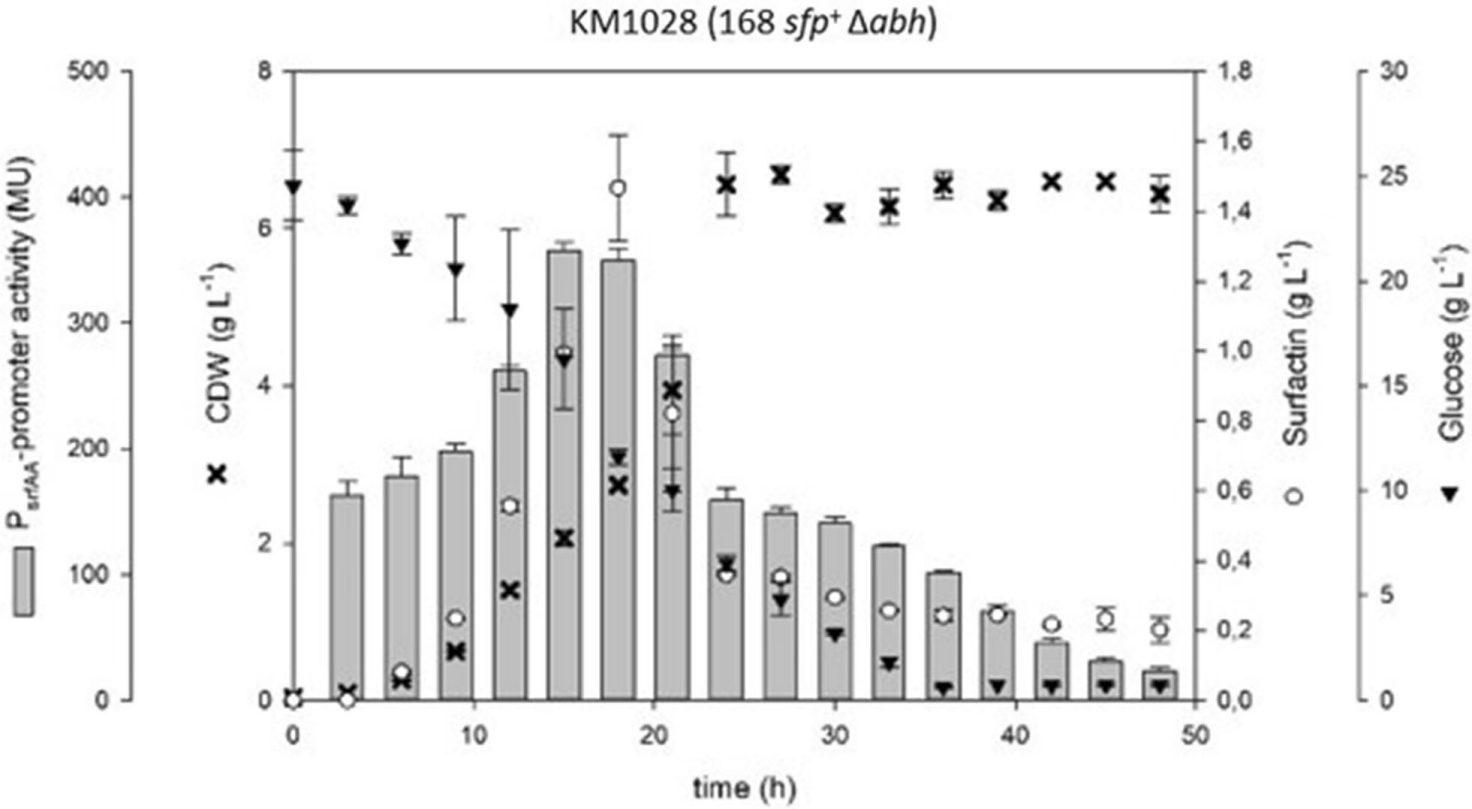
Time course of shake flask cultures of *B. subtilis* KM1028 (168 *sfp*^+^ Δ*abh*) cultivation displaying biomass (black crosses), surfactin (white circles) and glucose (black inverted triangles) concentrations in [g L^−1^] as well as P_*srfA*_ promoter activity in MU (grey bars) over time

Beside the impact of AbrB as transcriptional regulator, its homolog Abh has a synergistical effect. Moreover, both regulators are able to build both homomers and heteromers [4]. To get more insights about Abh impact on surfactin production, a Δ*abh* deletion mutant strain was constructed, called KM1028 (168 *sfp*^+^ Δ*abh*). As shown in Fig. 4, KM1028 exhibited a similar growth pattern compared to KM1016, with increased surfactin titers of up to 1.47 g L^−1^. Maximum promoter activity and growth factors can also be reviewed in Table 1.

### Effect of combined 3NA genetic features on surfactin production

**Fig. 5:**
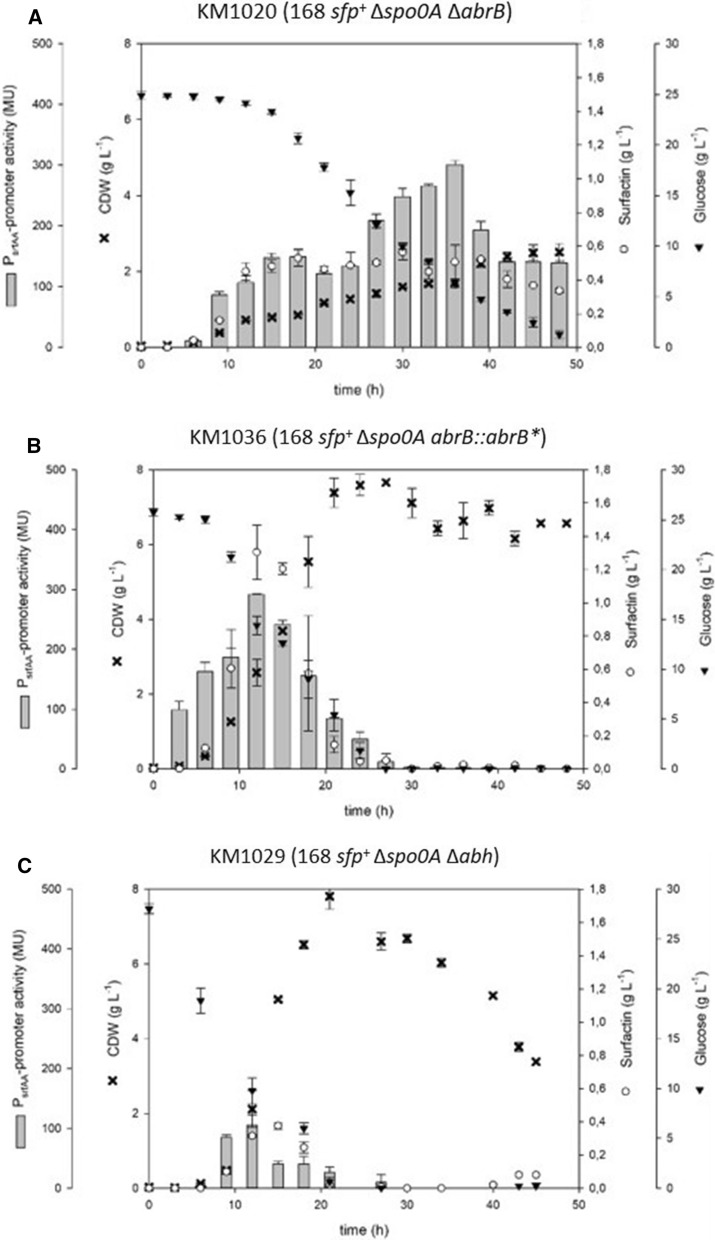
Time course of shake flask cultures of *B. subtilis* double mutant strains KM1020 (168 *sfp*^+^ Δ*spo0A* Δ*abrB*) (**A**), KM1036 (168 *sfp*^+^ Δspo0A abrB*) (**B**) and KM1029 (168 *sfp*^+^ Δ*spo0A* Δ*abh*) (**C**) displaying biomass (black crosses), surfactin (white circles) and glucose (black inverted triangles) concentrations in [g L^−1^] as well as P_*srfA*_ promoter activity (grey bars) in MU over time

Based on regulatory interconnections between gene products described before, *spo0A* deletion was combined with *abh* and *abrB* deletions as well as *abrB* elongation (abrB*). In this way, the detailed influence of 3NA genetic features (Δ*spo0A*, abrB*) on surfactin production could be investigated. Hence, combinatory mutant strains KM1020 (168 *sfp*^+^ Δ*spo0A* Δ*abrB*), KM1029 (168 *sfp*^+^ Δ*spo0A* Δ*abh*) and KM1036 (168 *sfp*^+^ Δ*spo0A abrB*::abrB*) were constructed. KM1020 exhibited a comparably low growth rate of 0.07 h^−1^. In contrast to KM1016, KM1020 reached surfactin concentrations of only 0.56 g L^−1^_,_ while maximum promoter activities of up to 301 MU were detected (Fig. 5A). Both kinetics of growth and promoter activity were comparable to single mutant strains KM1019 (168 *sfp*^+^ Δ*abrB*) and KM1043 (168 *sfp*^+^ *abrB*::abrB*).

Growth rates of KM1036 were higher compared to KM1016, but lower compared to KM1053. Regarding surfactin production, KM1036 reached maximum surfactin titers of 1.47 g L^−1^ and a maximum promoter activity of 292 MU (Fig. 5B).

The strain KM1029 performed like KM1018 in all aspects (Fig. 5C), with low surfactin titers and low promoter activity, but high growth rates compared to KM1016 (Table 1).

**Table 1:** Summary of surfactin production parameters for all mutant strains constructed in this study

Strain	Genotype	CDW_Srf,max_	Surfactin_max_	Μ = μ_overall_	Y_P/X_	Y_P/S_	P_*srfA*,max_
		g L^−1^	g L^−1^	h^−1^	g g^−1^	g g^−1^	MU
KM1016	168 *sfp*^+^	2.25	1.20	0.176	0.53	0.14	414
KM1018^a^	Δ*spo0A*	4.67	0.22	0.274	0.05	0.01	57
KM1019^a^	Δ*abrB*	2.77	1.25	0.080	0.45	0.08	351
KM1043^a^	*abrB*::abrB*	3.06	1.40	0.078	0.46	0.08	400
KM1028^a^	Δ*abh*	2.74	1.47	0.174	0.54	0.11	357
KM1020^a^	Δ*spo0A* Δ*abrB*	0.85	0.56	0.067	0.62	0.04	301
KM1036^a^	Δ*spo0A abrB*::abrB*	2.77	1.31	0.207	0.47	0.11	292
KM1029^a^	Δ*spo0A* Δ*abh*	5.05	0.38	0.205	0.07	0.02	105
KM1053	3NA *sfp*^+^	3.35	1.42	0.285	0.42	0.10	254

^a^Strains were constructed with KM1016 as output strain

### Comparative fed-batch bioreactor cultivation of *B. subtilis* 168 strain encoding 3NA features

**Fig. 6:**
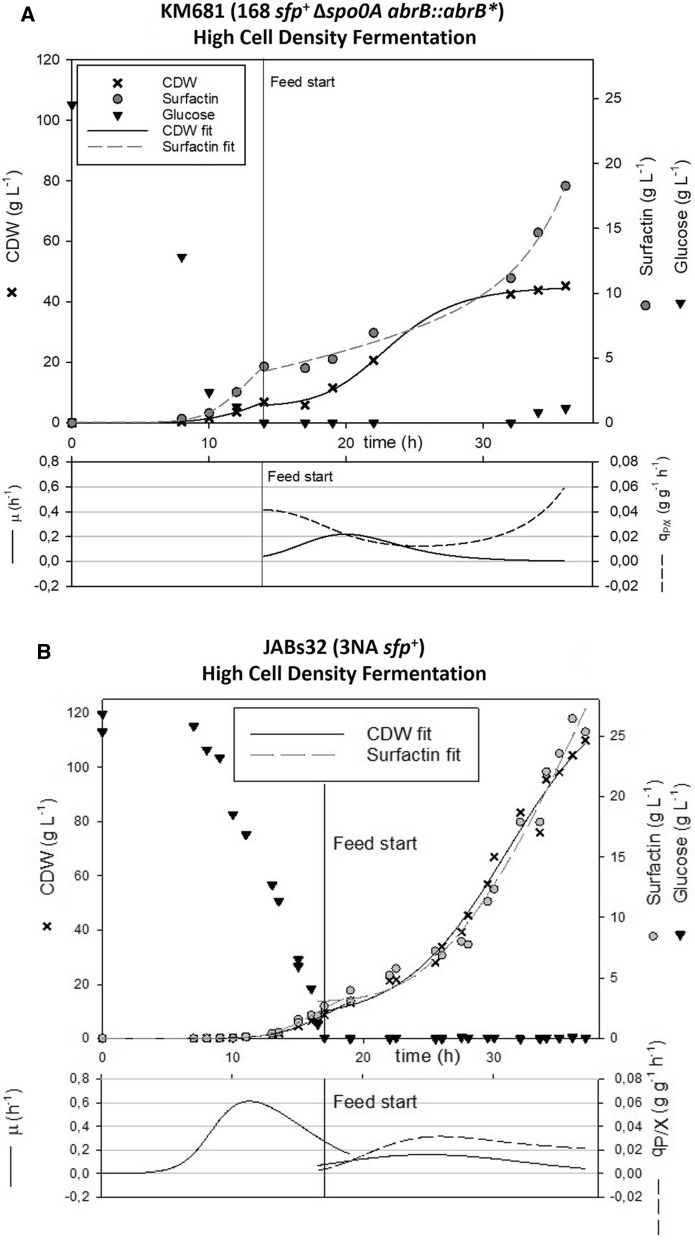
Comparison of fed-batch bioreactor fermentation of KM681 (168 *sfp*^+^ Δ*spo0A abrB*::abrB*) and JABs32 (3NA *sfp*^+^) (published by Klausmann et al. [15]). Shown are biomass concentration in [g L^−1^] (black crosses), surfactin concentration in [g L^−1^] (grey circles) and glucose concentration in [g L^−1^] (black inverted triangles) as well as growth rate (solid line) and specific productivity (dotted line) over time

To compare surfactin production capabilities of the mutant strain KM681 (168 *sfp*^+^ Δ*spo0A abrB*::abrB*) to that of the JABs32 (3NA *sfp*^+^) reference process [15], a fed-batch fermentation in a 30 L bioreactor was conducted as described by Klausmann et al. [15]. At the end of batch phase after 14 h, KM681 reached a CDW of 6.91 g L^−1^ and the fed-batch-phase was induced (Fig. 6A). This phase lasted for 22 h and at the end a maximum CDW of 45.21 g L^−1^ was measured. This corresponds to a total biomass of 836.4 g in 18.5 L of culture medium. Surfactin titers after fed-batch phase reached a maximum of 18.27 g L^−1^, meaning a total of 338 g. Accordingly, a substrate-to-product yield Y_P/S_ of 0.121 g g^−1^ and a product-per-biomass yield Y_P/X_ of 0.404 g g^−1^ was reached after fed-batch cultivation. Compared to strain JABs32 [15] (Fig. 6B) these parameters represent a 49% reduction in biomass concentration and a 32% reduction in surfactin concentration. However, compared to previously presented results for JABs24 (168 *sfp*^+^) [15], an increase about 292% was achieved. In terms of their specific productivity q_P/X_ yields JABs32 exhibited a slow decline over time while KM681 displayed a significant increase during the second half of fed-batch fermentation process (Fig. 6A, B). Growth rates of KM681 were continually declining over time after reaching µ_max_ of 0.22 after 22 h, indicating a stationary phase towards the end of cultivation with a µ_min_ of 0.004 after 36 h. In contrast, JABs32 did not enter stationary phase over the time course of its fermentation process and showed a µ_max_ of 0.16 after 25 h, though it also displayed declining growth rates over time and reached a µ_min_ of 0.04 after 37 h.

## Discussion

**Fig. 7:**
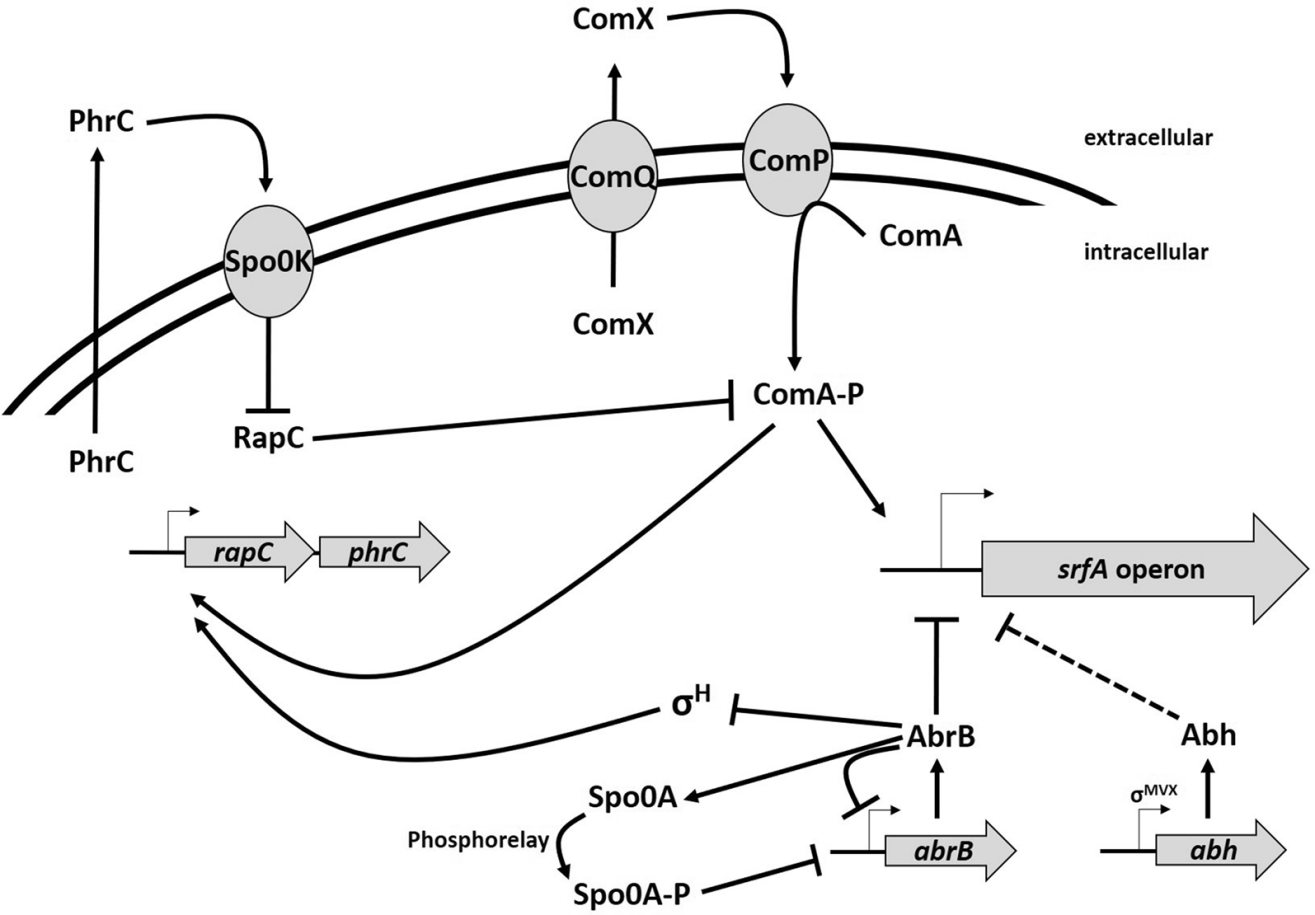
Overview of a part of the regulatory mechanisms and their interplay that influence the gene expression of the surfactin-forming *srfA* operon in *B. subtilis*. Global cellular differentiation processes are involved, such as ComX-mediated competence development, Spo0A dependent initiation of the sporulation and regulatory crosstalk of the AbrB regulator

In this work, mutations in the *abrB* and *spo0A* genes found in *B. subtilis* strain 3NA were introduced into strain KM1016, an *sfp*^+^ derivative of *B. subtilis* 168. The use of single and combinatory mutant strains was intended to answer the overall question of the extent to which mutations in the 3NA strain influence surfactin production capacity, as described by Klausmann et al. [15]. Additionally, *abrB* homologue *abh* was investigated for its capability of negating the effects of *abrB* deletion or inactivation. Strains KM1016 (168 *sfp*^+^) and KM1053 (3NA *sfp*^+^) both revealed similar growth behavior and surfactin production comparable to their progenitors JABs24 [12] and JABs32, respectively [15]. When compared, reference strain KM1053 was found to produce more surfactin (1.42 g L^−1^) than KM1016, even though lower P_*srfA*_ promoter activity (254 MU vs. 414 MU) was detected using the Miller assay. These results indicate that surfactin production is not only dependent on transcriptional activity. One bottleneck could be the availability of precursor molecules, as studies have shown that improved metabolic pathways of precursors increased surfactin titers [39]. Another bottleneck is the multiply regulated promoter region of the *srfA* operon. Several studies have demonstrated that an increase in promoter activity leads to enhanced surfactin titers [13, 32, 37, 39], although in some cases *B. subtilis* strains exhibited reduced titers [37]. However, in our study, the results of KM1016 (168 *sfp*^+^) show a correlation between P_*srfA*_ promoter activity and cell growth during exponential growth. In this phase, quorum sensing seems to be a major influencing factor, which can be comprehended by the accumulation of the ComX pheromone [7]. In the subsequent regulatory crosstalk, ComX indirectly initiates the activation of ComA [10], which is a positively acting regulator for the *srfA* operon [5, 23]. After reaching the transition to stationary phase, a drastic decline in P_*srfA*_ promoter activity was observed, indicating a switch in the regulatory mechanisms for surfactin formation. In this context, especially the global regulator AbrB seems to have a negative effect on the expression of the *srfA* operon. Strains KM1019 (Δ*abrB*) and KM1043 (*abrB*::abrB*) showed overall increased P_*srfA*_ promoter activities, suggesting that the AbrB regulator is a negating factor for surfactin production, especially during stationary phase. Confirmations were provided by the observations of KM1018 (Δ*spo0A*), which showed drastically reduced P_*srfA*_ activity. Since Spo0A is a repressor of *abrB* gene expression, deletion of *spo0A* leads to overexpression of *abrB* [24], resulting in the observed reduction in P_*srfA*_ promoter activity [2, 30, 35]. This complex regulatory interplay is summarized in Fig. 7.

In this context, the impact of AbrB and its elongation from 3NA reference strain were analyzed in strains KM1019 (Δ*abrB*) and KM1043 (*abrB*::abrB*). Interestingly, both mutant strains exhibited similar, reduced growth rates and almost linear cell growth compared to the KM1016 reference strain (Table 1). An associated agglutination could be the result of deregulated target genes of AbrB regulon such as biofilm-associated *epsA*-*O* operon [4]. However, KM1043 reached its maximum CDW faster than KM1019 and had a higher glucose consumption. Accordingly, glucose was consumed after 36 h in KM1043, while KM1019 had about 1.2 g L^−1^ glucose left in the medium after 48 h. Surfactin titers were slightly different between these strains. While KM1019 had maximum concentrations of 1.25 g L^−1^ which are similar to KM1016, a surfactin production of 1.4 g L^−1^ was detected for KM1043 that was comparable to KM1053. In addition, both strains exhibited relatively high P_*srfA*_ promoter activities during the entire cultivation. This promoter expression pattern differed significantly from previously described reference and mutant strains, which exhibited a strong decrease in promoter activity as well as surfactin concentrations after the first 27 h of cultivation or after their maximum CDW was reached, respectively. These observations suggest that elongation of AbrB plays a partially modulating role in respect to regulator activity, although further studies need to address this issue in detail. Altogether, the results indicate that surfactin production is tightly coupled to cell growth during the exponential phase. As growth rates decreased in KM1019 and KM1043, surfactin titers decrease as well after about 39 h.

Abh was described as an AbrB homolog which is able to bind some promoter regions previously described as AbrB regulated [4, 22, 31]. In this context, Chumsakul et al. [4] had shown that AbrB and Abh are able to form both homomers and heteromers. This led to the assumption that Abh might also have some influence on surfactin production in *B. subtilis* as previously described by Chumsakul et al. [4]*.* The strains KM1028 (Δ*abh*) and KM1029 (Δ*abh* Δ*spo0A*) were constructed to test this hypothesis. In the case of the double mutant KM1029, surfactin production and P_*srfA*_ promoter activity were approx. twice as high as Δ*spo0A* mutant KM1018. Cell growth, however, was similar for both strains. A comparison of KM1028 to the reference strain KM1016 showed no differences in growth behavior and promoter activity. Nevertheless, a slight increase in surfactin titer of 1.47 g L^−1^ was measured compared to KM1016 (1.2 g L^−1^). In combination with results of KM1029, the conclusion is that Abh has a minor negative effect on surfactin promoter activity and surfactin production.

In terms of growth behavior, strain KM1018 (Δ*spo0A*) exhibited a similar growth behavior as 3NA reference strain KM1053. This observation indicates that the nonsense mutation in the *spo0A* gene of JABs32 and KM1053 led to their increased growth rate compared to JABs24 and KM1016. After reaching stationary phase, the CDW of KM1018 decreased drastically after about 30 h. This phenomenon was not observed in KM1053, which encodes a mutated *spo0A* version and an elongated *abrB* version (abrB*). Accordingly, abrB* could be the reason for the altered growth behavior during stationary phase compared to KM1018. Based on the derepressed *abrB* gene expression in KM1018, a deletion or inactivation of *abrB* would have a reversible effect on a Δ*spo0A* phenotype. This was shown in combinatory mutant strains KM1020 (Δ*spo0A*; Δ*abrB*) and KM1036 (Δ*spo0A*; *abrB*::abrB*). Compared to reference strains and other single mutant strains, KM1036, which encodes the main genetic differences between 168 and 3NA strain, demonstrated similar properties to the reference strain KM1053 in all parameters, namely growth behavior, surfactin production and P_*srfA*_ promoter activity. In contrast, KM1020 exhibited slow, linear cell growth to a maximum CDW of only 2.5 g L^−1^ at the end of cultivation combined with comparably low surfactin titer of 0.54 g L^−1^. Accordingly, the assumption is that both *spo0A* deletion and *abrB* elongation are crucial for 3NA phenotype as promising surfactin production strain. Combined with previous results of KM1043, the AbrB elongation seems to have a different effect on surfactin production than a deletion thereof. Accordingly, it is reasonable to assume that deletion of *spo0A* increased AbrB expression resulting in an enhanced repression of surfactin production. The elongation of *abrB* as well as its deletion would reverse or modify this effect, respectively. However, differences between both *abrB* mutant strains in respect of surfactin production and growth behavior indicate that the elongated AbrB version could exhibit residual activity. In consequence, the AbrB regulon would still be active in a modified or reduced way. Further investigations could help to identify putative alterations in the availability of precursor molecules for surfactin production between KM1020 and KM1036. Furthermore, proteomic and transcriptomic analyses will help to verify in detail the effect of *abrB* elongation compared to deletion mutants and their regulatory crosstalk with Spo0A. Future studies should also look into the effect of the deletions on surfactin synthesis decoupled from quorum sensing regulation. For this purpose, a constitutive promoter should be used to express the *srfA* operon in the presented mutant strains as has been demonstrated previously in Willenbacher et al. [37] and Vahidinasab et al. [33].

A subsequent decline in surfactin after the exponential growth phase was also observed by Klausmann et al. [15]. One explanation is the limitation of specific nutrients in the cultivation medium, as Willenbacher et al. [38] were able to show a comparable decrease in surfactin in cultivations with 40 g L^−1^ glucose, while stabilized surfactin concentrations were detected with 6 g L^−1^ glucose. Accordingly, surfactin could be degraded for nutrients or be involved in the uptake of trace elements. This hypothesis is consistent with the overall growth rates µ determined (Table 1). The slower-growing mutant strains consumed fewer nutrients, resulting in delayed nutrient limitations. Accordingly, the surfactin concentration reached a larger plateau compared to the reference strains (µ_KM1016_ = 0.176 h^−1^, µ_KM1053_ = 0.285 h^−1^). This was the case for KM1019 (µ = 0.080 h^−1^), KM1043 (µ = 0.078 h^−1^) and KM1020 (µ = 0.067 h^−1^).

As reported previously, *B. subtilis* strain JABs32 exhibits promising surfactin production rates [15]. Surfactin titers of up to 26 g L^−1^ are achievable using a fed-batch process. Based on these observations, a surfactin-forming derivative of *B. subtilis* 168 strain, KM681, encoding both *spo0A* deletion and *abrB* elongation without *lacZ* reporter gene, was used in fed-batch bioreactor cultivations and production parameters were compared to the reference process with strain JABs32 (3NA *sfp*^+^) and JABs24 (168 *sfp*^+^) published by Klausmann et al. [15]. In this fermentation, KM681 was able to reach high cell densities of up to 45 g L^−1^ CDW. Surfactin titers reached up to 18.27 g L^−1^ at the end of fermentation. This represents a decrease of about 32% compared to the cultivation of JABs32 and an increase of about 292% compared to JABs24 as presented by Klausmann et al. [15]. These results showed that construction of a high cell density *B. subtilis* strain for surfactin production is feasible by elongation of AbrB in combination with deletion of *spo0A.*

This work has shown that the positive impact of a high cell density fermentation process can be achieved by deletion of the *spo0A* gene coupled with the elongation of AbrB. It has also demonstrated that deletion and elongation of *abrB* had different effects on strain growth and surfactin production and therefore that the elongation does not solely lead to an inactivation but rather to a change in AbrB regulator activity.

## Conclusions

*Bacillus subtilis* 3NA is already established as a production strain for high cell density fermentations with promising surfactin production capabilities. Investigations of the notable mutations concerning the global regulators Spo0A and AbrB showed that the beneficial 3NA phenotype for surfactin production is based on both genetic modifications, namely the inactivation of *spo0A* and elongation of *abrB*. While a *spo0A* deletion resulted in fast growing *B. subtilis* strains, *abrB* elongation was associated with high surfactin production capacities. A compensatory effect on surfactin production was only found for the *abrB* elongation, while deletion of *abrB* and the homologous *abh* in combination with *spo0A* deletion showed varying and only slightly improved surfactin production rates, suggesting an altered regulator activity of elongated AbrB version. With this knowledge, it is possible to easily generate non-sporulating strains of *B. subtilis* for various high cell density bioprocesses for the production of biosurfactants, especially surfactin.

## Methods

### Chemicals, materials and standard procedures

All chemicals were acquired from Carl Roth GmbH & Co. KG (Karlsruhe, Germany) if not mentioned otherwise. Standard molecular methods were conducted as described before by Sambrook et al. [28]. Chromosomal DNA and plasmid DNA were purified by application of innuPREP Bacteria DNA Kit and innuPREP Plasmid Mini Kit, respectively (Analytik Jena AG, Jena, Germany). All primers used for PCR reactions were synthesized by Eurofins Genomics (Ebersberg, Germany). DNA fragments were amplified by polymerase chain reactions using Phusion High-Fidelity DNA Polymerase (New England BioLabs, Frankfurt am Main, Germany). PCR reactions were performed with thermo cycler (prqSTAR 96X VWR GmbH, Darmstadt, Germany). Amplified PCR products were extracted with QIAquick PCR & Gel Cleanup Kit (QIAGEN GmbH, Hilden, Germany).

### Bacterial strains and conditions of cultivation

Strains used for experiments were listed in Table 2. The first precultures were performed in LB medium with 10 g L^−1^ tryptone, 5 g L^−1^ yeast extract and 5 g L^−1^ NaCl. The second preculture was inoculated in the respective cultivation medium of the main culture. Main cultivations were inoculated with an initial OD_600_ of 0.1 and were performed in 1 L baffled shake flasks using 100 mL synthetic medium [38] containing 27.5 g L^−1^ glucose × H_2_O, 7.12 g L^−1^ Na_2_HPO_4_, 4.08 g L^−1^ KH_2_PO_4_, 6.61 g L^−1^ (NH_4_)_2_SO_4_, 0.197 g L^−1^ MgSO_4_ × 7 H_2_O and 1 mL L^−1^ trace element solution (TES). TES contained 2.35 g L^−1^ Na_3_citrate, 0.78 g L^−1^ CaCl_2_, 1.11 g L^−1^ FeSO_4_ and 0.16 g L^−1^ MnSO_4_ × H_2_O. The pH of the media used for shake flask cultivations was adjusted to 7.0.

**Table 2:** List of bacterial strains and plasmids used in this study

Strains and plasmids	Genotypes or descriptions	References
Strains		
*Escherichia coli*		
JM109	*mcrA recA1 supE44 endA1 hsdR17*	[40]
	(*r*_K_^−^*m*_K_^+^) *gyrA96 relA1 thi* Δ(*lac-proAB*)	
	*F*′[*traD36 proAB*^+^ *lacI*^q^ *lacZ* Δ*M15*]	
*Bacillus subtilis*		
JABs24	*trp*+ Δ*manPA sfp*^+^	[9]
JABs32	Δ*manPA sfp*^+^ spo0A3 abrB*	[15]
KM1053	Δ*manPA sfp*^+^ spo0A3 abrB*	This study
	*amyE*::[P_*srfA*_*-lacZ*, *spcR*]	
KM1016	168 *trp*^+^ Δ*manPA sfp*^+^	[12]
	*amyE*::[P_*srfA*_*-lacZ*, *spcR*]	
KM1019	168 *trp*^+^ Δ*manPA sfp*^+^ Δ*abrB*::*loxP*	This study
	*amyE*::[P_*srfA*_*-lacZ*, *spcR*]	
KM1043	168 *trp*^+^ Δ*manPA sfp*^+^ *abrB*::abrB*-*cat*	This study
	*amyE*::[P_*srfA*_-*lacZ*, *spcR*]	
KM1018	168 *trp*^+^ Δ*manPA sfp*^+^	This study
	Δ*spo0A*::*loxP-ermC-loxP*	
	*amyE*::[P_*srfA*_*-lacZ*, *spcR*]	
KM1020	168 *trp*^+^ Δ*manPA sfp*^+^	This study
	Δ*spo0A*::*loxP-ermC-loxP* Δ*abrB*::*loxP*	
	*amyE*::[P_*srfA*_*-lacZ*, *spcR*]	
KM1036	168 *trp*^+^ Δ*manPA sfp*^+^	This study
	Δ*spo0A*::*loxP-ermC-loxP abrB*::abrB*-*cat*	
	*amyE*::[P_*srfA*_*-lacZ*, *spcR*]	
KM1028	168 *trp*^+^ Δ*manPA sfp*^+^ Δ*abh*::*loxP*	This study
	*amyE*::[P_*srfA*_*-lacZ*, *spcR*]	
KM1029	168 *trp*^+^ Δ*manPA sfp*^+^	This study
	Δ*spo0A*::*loxP-ermC-loxP* Δ*abh*::*loxP*	
	*amyE*::[P_*srfA*_*-lacZ*, *spcR*]	
KM681	168 *trp*^+^ Δ*manPA sfp*^+^	This study
	Δ*spo0A*::*loxP-ermC-loxP abrB*::abrB*-*cat*	
BKE24220	*trpC2 spo0A*::*erm*	*Bacillus* Genetic Stock Center
BKE14480	*trpC2 abh*::*erm*	*Bacillus* Genetic Stock Center
BKE00370	*trpC2 abrB*::*erm*	*Bacillus* Genetic Stock Center
Plasmids		
pKAM446	*ori*_pUC18_*, bla*, *rop*, *ermC*,	[12]
	*amyE*′-[*ter*-P_*srfAA*_-*lacZ*, *spcR*]-′*amyE*	
pJOE7644.2	*ori*_pUC18_, *bla*, P_*manP*_-*manP*, *spcR*, ′*manR-ctaO*′	[21]

All cultivations were conducted as biological triplicates and were performed at 37 °C and 120 rpm in an incubation shaker (Innova 44®R, Eppendorf AG, Hamburg, Germany). The fermentation process was conducted as described by Klausmann et al. [15].

### Construction of mutant strains

All primers for the strain construction were listed in Table 3. The mutant strains used in this study are listed in Table 2 and were derived from *B. subtilis* strains KM1016, a derivative of JABs24 [12] and KM1053, generated from strain JABs32 [15]. Gene deletions were integrated by transformation of linear DNA fragments amplified by PCR from BKE strains BKE24220 (*trpC2 spo0A::erm*), BKE14480 (*trpC2 abh::erm*) and BKE00370 (*trpC2 abrB::erm*) [16]. The elongation of *abrB* locus (abrB*) associated with *cat* resistance marker was amplified from *B. subtilis* strain IIG168-13. Homologous up- and downstream sequences of the respective target gene allowed the integration of amplified DNA fragments into the bacterial chromosome. Transformation of natural competent *B. subtilis* strains was performed according to the “Losick protocol”. Mutants were selected on LB agar plates containing erythromycin (5 µg mL^−1^), spectinomycin (150 µg mL^−1^) or chloramphenicol (5 µg mL^−1^).

**Table 3:** Primers used in this study

Name	Sequence 5′–3′	Purpose
s1009	CTGCCGTTATTCGCTGGATT	Amplification of *amyE* locus
s1010	AGAGAACCGCTTAAGCCCGA	
s1055	GGCGCCAAATGAGCTTTAATGGA	Amplification of abrB*-cat from *B. subtilis* IIG168-13 and *abrB*::*erm* from BKE00370
s1056	TGACCGCTGTCAGGGCTTTT	
s1070	ACTGCGATTTTTGGGGGTGT	Amplification of *abh*::*erm* from BKE14480
s1071	TCTCATATGACCACCTGCCG	
s1221	CGATATGGACACAAAGAAACC	Amplification of *spo0A*::*erm* from BKE24220
s1222	CAATGACTGAAACTTATACGCTTG	

An additional transformation of selected mutant strains with pJOE7644.2 was performed for removal of erythromycin resistance cassette resulting in markerless gene deletions.

The chromosomal DNA loci of final mutant strains were checked for correctness by sequencing (Eurofins Genomics Germany GmbH, Ebersberg, Germany).

### Analytical methods

Glucose analysis was conducted using the enzyme assay kit from R-Biopharm (R-Biopharm AG, Darmstadt, Germany, Cat no. 10148261035). Ammonia concentration was analysed with a photometric ammonia test kit (Merck KGaA, Darmstadt, Germany, Cat no. 1.14752.0001).

For the calculation of cell dry weight (CDW) a factor of 0.322 from OD_600_ was used for KM1016 and derived mutant strains and 0.372 for KM1053 [15]. To determine the correction factors, samples of the respective strain were pelleted by centrifugation, washed three times with 0.9% (w/v) saline solution and dried at 110 °C for 48 h. Afterwards, the samples were weighed and from a linear retention curve, the slope was determined as the correlation factor of OD_600_ and CDW.

The β-galactosidase assay was performed as previously described by Hoffmann et al. [12]. In brief, 100 µL of the cell suspension was mixed with 900 µL Z-Buffer followed by addition of 10 µL toluol. After an incubation for 30 min at 37 °C and 750 rpm, 200 µL of 20 mM *ortho*-nitrophenylgalactopyranoside (ONPG) was added. When the mixture turned yellow, the reaction was stopped by using 500 µL of 1 M sodium carbonate solution. After sedimentation of precipitations by centrifugation, 250 µL of the reaction mixture were used for measurement in a microtiter plate. Absorbance was measured at 420 nm and 550 nm. Miller Units (MU) were calculated with Eq. (1):

1 $$ {\text{MU}} = 1000 \times \frac{{\left( {OD420 \;{\text{nm}} - \left( {1.75 \cdot OD550 \;{\text{nm}}} \right)} \right)}}{{t \cdot v \cdot OD600 \;{\text{nm}}}}. $$

Surfactin analysis was conducted as described by Geissler et al. [8] by HPTLC analysis (CAMAG AG, Muttenz, Switzerland). In brief, 2 mL of a cell-free sample was extracted three times with 2 mL of chloroform:methanol (2:1). The organic phase was pooled and dried using a rotary evaporator (RVC2-25 Cdplus, Martin Christ Gefriertrocknungsanlagen GmbH, Osterode am Harz, Germany) at 40 °C and 10 mbar for 45 min. The pellet was resuspended in 2 mL methanol. A surfactin standard (Sigma-Aldrich, Seelze, Germany) and the sample were applied in a range of 30 to 600 ng and developed with a mobile phase of chloroform:methanol:water (65:25:4) over a migration distance of 60 mm. The plate was then analysed at 195 nm for surfactin detection. To quantify surfactin production, a surfactin standard from Sigma Aldrich (St. Louis, USA) was used.

### Bioreactor fermentation

Fed-batch bioreactor fermentation processes were performed as described before by Klausmann et al. [15] with small variations. An overnight culture in LB medium was inoculated from a glycerol stock and incubated at 37 °C for 13 h at 120 rpm. A second preculture in HCDM containing 25 g L^−1^ glucose was inoculated from the overnight culture to an OD_600_ of 0.1 and incubated for 8 h at 37 °C and 120 rpm. Therefrom, a 30 L bioreactor with 12 L HCDM was inoculated to an OD_600_ of 0.1.

The fermentation was set to a temperature of 37 °C, a pH of 7.0, a minimal pO_2_ of 70% and a foam centrifuge as well as antifoaming agent Contraspum 300 (Zschimmer & Schwarz GmbH, Lahnstein, Germany) was employed to prevent overfoaming of the bioreactor. The pH was controlled by 4 M H_3_PO_4_ and 20% (v/v) NH_3_ solutions. A foam trap was installed using a 25 L container filled with 3 L of water and 20 mL of Contraspum 300.

The batch phase was run overnight and the fed-batch was started the next day as soon as glucose was depleted after 12 h. The feed solution I consisted of 5 L 50% (w/w) glucose, 12 g L^−1^ MgSO_4_ and 120 mL L^−1^ TES, while Feed II was comprised of 1.5 L 396 g L^−1^ (NH_4_)_2_HPO_4_. The initial feed rate was calculated as described by Klausmann et al. [15] and the growth rate was set to 0.1.

### Data analysis

The yield of biomass per substrate (Y_X/S_), product per biomass (Y_P/X_), growth rate µ and specific productivity (q_P/X_) were determined using the equations shown below as previously described by Klausmann et al. [15]. Glucose and ammonia concentrations, as well as CDW and surfactin titers were plotted for every sampling time point. Acetate concentration was determined at the start, as well as in the middle and at the end of the fed-batch phase to rule out negative effects on growth or surfactin production [12].

2 $$ Y_{X/S} = \left. {\frac{X}{\Delta S}} \right|_{{X = X_{max} }} , $$

3 $$ Y_{P/S} = \left. {\frac{P}{\Delta S}} \right|_{{P = P_{max} }} , $$

4 $$ Y_{P/X} = \left. \frac{P}{X} \right|_{{P = P_{max} }} , $$

5 $$ q_{P/X, overall} = \frac{{P_{max} }}{{X_{{P_{max} }} \cdot \Delta t}}, $$

6 $$ q_{P/X} \left( t \right) = \frac{\Delta P}{{X \cdot \Delta t}}, $$

7 $$ \mu_{overall} = \frac{{\ln \left( {CDWx_{max2} } \right) - \ln \left( {CDWx_{t01} } \right)}}{\Delta t}. $$

The fitted curves shown in Fig. 6 were derived using scientific graphing and data analysis software (SigmaPlot, Systat Software Inc., San Jose, CA). Therefore, a dynamic fit function of SigmaPlot14 was used including a 4-parameter logistic fit. The generated fit values were applied to calculate growth rate µ and specific productivity q_P/X_.

## Data Availability

All raw data and biological material are saved in the institute of Food Science and Biotechnology, Department of Bioprocess Engineering (150k), University of Hohenheim, Fruwirthstraße 12, Stuttgart 70599, Germany. In case of requirement, please contact the corresponding author for any detailed question.
